# Seminal gold content in healthy fertile men in India

**DOI:** 10.4103/0974-7788.72490

**Published:** 2010

**Authors:** Vinod Jain, Anurag Rai, Samir Misra, K.M. Singh

**Affiliations:** *Department of General Surgery, C.S.M.Medical University, (Erstwhile - King George Medical University), Lucknow, Uttar Pradesh, Lucknow, India*

**Keywords:** Atomic absorption spectrophotometry, fertile men, seminal gold, sperm

## Abstract

**Objective:**

Since centuries Ayurveda, mentions the role of gold in the treatment of male infertility ‘*Swarna Bhasma*’ (Ash of gold) has been used with good results by Ayurvedic practitioners in the treatment of infertility. Hence, a study was planned to estimate gold in whole semen by atomic absorption spectrophotometry.

**Materials and Methods:**

Whole semen from 11 healthy males of proved fertility from Lucknow (India) was analyzed for gold content by Atomic Absorption spectrophotometry at wavelength 242.8 nm with Hollow Cathode Gold Lamp. Prior to analysis, all the samples were subjected to digestion procedure, achieved by treating them with mixture of concentrated Nitric acid and concentrated Perchloric acid in 6: 1 ratio.

**Observation:**

On analysis all semen samples were found to contain gold ranging from 0.36 to 1.98 μg/ml with a mean value of 0.88 μg/ml and a standard deviation of 0.51 μg/ml.

**Conclusion:**

In the present study, gold was estimated after complete digestion (oxidation of organic matters; hence, whatever amount of gold detected, denotes the levels in seminal plasma as well as the sperm itself) in whole semen (seminal plasma and sperm). It seems that the hypothesis made for presence of gold in sperm might be true. However, the literature available in this connection is very scanty and further studies are needed for scientific documentation of gold in male infertility.

## INTRODUCTION

The *mileu* surrounding sperm plays an important role in its vital activities.[1,2] From time to time various inorganic metals have been documented to have beneficial as well as adverse effects on the human sperm.[1–8] Since centuries Ayurveda, the ancient medicine of India, mentions the role of gold in the treatment of male infertility.[9,10] ‘*Swarna Bhasma*’ (Ash of gold) has been used with good results by Ayurvedic practitioners in the treatment of infertility.[11–13] However, no scientific document that confirms the role of gold.[14] An earlier study Skandhan[15] reported the presence of gold in human semen plasma in two of the fourteen cases studied [Table 1]. The cause for the presence of gold in those two samples was thought to be leakage from sperms. With this background, a study was planned to estimate gold in whole semen by atomic absorption spectrophotometry.

**Table 1:** Semen characteristics and gold content in present study

Volume	L.T. (min)	Sperm counts (mill/ml)	Motility (%)	Gold content μg/ml
2.0	15	104	80	0.36
3.5	20	112	80	1.92
3.0	20	112	75	0.96
2.5	10	98	75	1.68
4.0	15	108	85	0.66
4.0	10	108	80	0.66
3.0	10	154	90	0.66
2.5	15	96	90	0.36
2.5	10	142	85	0.78
2.0	15	118	85	0.32
3.0	10	164	75	0.36
Blank (Solvent Distilled water)				N.D.

L.T. – Liquefaction time; mill./ml – millions/ml; N.D. – Not detected.

## MATERIALS AND METHODS

Eleven healthy male volunteers from Lucknow of age 26–40 years, with proven fertility (having at least two offspring), were selected for the present study (after having written informed consent). None of them had ever been admitted in hospital for any disease and none had ever received gold therapy in any form. Manual semen analysis was used using World Health Organization reference values.[16–18]

We had not used Computer-Assisted Sperm Analysis (CASA) (refers to a semi-automated technique used to individualize and digitalize static and dynamic sperm images) because when used clinically, CASA has not been documented to give a more accurate prognosis or to affect treatment as compared with a manual semen analysis. For assessment of the quality of sperms we used forward movement on a five-point scale. A rating of zero signifies no motility; 1 denotes sluggish or non-progressive movement; 2 refers to sperm moving with a slow, meandering forward progression; 3 signifies sperm moving in a reasonably straight line with moderate speed and scale 4 indicates sperm moving in a straight line with high speed. Their semen samples were collected after abstinence by masturbation between 9 a.m. to 11 a.m. During collection and processing, none of the participants were wearing gold rings. The semen was allowed to liquefy at room temperature and after noting its physical characteristics, sperm counts and their motility, the samples were immediately transferred to deep freezer till further processing.

All the glassware used in present study was of Corning variety to prevent metal contamination. They were rinsed with heated 6 M Nitric acid three times and kept dipped in acid overnight, then they were thoroughly rinsed with distilled water followed by double and triple distilled water and finally heated dry in hot oven.

For estimation of gold, the samples were transferred in ice cooled box to Industrial Toxicological Research Center, Lucknow (India). There the samples were transferred to 100 ml conical flasks after accurate measurement of their volume. Ten ml of a mixture of Nitric acid (BDH-AR) 16 N and concentrated per-chloric acid (BDH-AR) in ratio of 6:1 was added to the semen sample. The sample was left overnight for complete digestion to occur. The next day, the mixture was evaporated gently at 70°C till all the acid was removed. The residue left behind was cooled and dissolved in 6 ml of N/10 HCl. Gold levels were measured by atomic absorption spectrophotometry under the following standard conditions, to give results in μg/ml. Readings were made at a wave length of 242.8 nm. The light source was a hollow Cathode Gold Lamp and the oxidizing air acetylene flame (Blue) was the flame used.

A stock solution of the strength of 1000 g gold/ml made from gold Chloride and standard concentrations of 0.1 and 3.0 μg/ml were used.

While analyzing the samples, the standard solution was analyzed at the beginning and end of the run and also periodically in between the samples to confirm the linear working of the instrument. Blank (solvent) was run after every sample and standard solution as well to verify the baseline variability.

For assessment of the quality of sperms we used movement (forward) on a five-point scale. Scale 4 indicates sperm moving in a straight line with high speed.

## RESULTS

Gold was found in all the digested semen samples and it ranged from 0.36 to 1.92 μg/ml. The mean ± SD was 0.88 ± 0.61 μg/ml. The results of present study are given in Table 1.The semen volume collected ranges from 2 to 4 ml, liquefaction time ranges from 10 to 20 min. sperm count ranges from 96 to 112, motility ranges 75 to 90. The color was white and viscosity was 0 in case of all samples. Microscopic examination shows no normality.

## DISCUSSION

The presence of gold in the body is thought to be a contaminant from environmental elements like sea water and total human body content of gold is calculated to be 0.1 mg. Oser[17] and Bondani *et al*.,[18] considered semen to be a source of excretion for electrolytes Although this may not be true for gold. Gold has been claimed to have beneficial effect on testicular function and sperm (*Ras Tantra Sar and Siddha Prayog Sangraha*).

In the present study, gold was estimated after complete digestion (oxidation of organic matters; hence, whatever amount of gold detected, denotes the levels in seminal plasma as well as the sperm itself) in whole semen (seminal plasma and sperm). The mean value of semen gold was found to be 0.88 μg/ml (SD ± 0.51), which is quite high when compared with the results of Skandhan.[15] Here it must be noted that Skandhan in his study had not included the sperm and did not mention about the digestion procedure (i.e. to convert all organically bound gold into inorganic forms which is the detectable form), which could be the possible cause for the high values of gold in our study. It seems that the hypothesis made for presence of gold in sperm might be true. However, the literature available in this connection is very scanty and further studies are needed for scientific documentation of gold in male infertility.
